# Phenotypic and proteomic analysis of plasma extracellular vesicles highlights them as potential biomarkers of primary Sjögren syndrome

**DOI:** 10.3389/fimmu.2023.1207545

**Published:** 2023-07-17

**Authors:** Juliette Ferrant, Adeline Pontis, François Zimmermann, Florent Dingli, Patrick Poullet, Damarys Loew, Karin Tarte, Erwan Dumontet

**Affiliations:** ^1^ Pôle Biologie, Centre Hospitalier Universitaire de Rennes, Rennes, France; ^2^ UMR, Université Rennes, INSERM, Établissement Français du Sang, Rennes, France; ^3^ Département de Médecine Interne et Immunologie Clinique, Centre Hospitalier Universitaire de Rennes, Rennes, France; ^4^ Institut Curie, PSL Research University, CurieCoreTech Mass Spectrometry Proteomics, Paris, France; ^5^ Institut Curie, PSL Research University, INSERM, Mines Paris Tech, Bioinformatics core facility (CUBIC), Paris, France

**Keywords:** Sjögren syndrome, extracellular vesicles, biomarker, proteomic, systemic lupus erythematosus

## Abstract

Sjögren syndrome (SjS) is an autoimmune disease characterized by the destruction of the exocrine gland epithelia, causing a dryness of mucosa called sicca symptoms, and whose main life-threatening complication is lymphoma. There is a need for new biomarkers in this disease, notably diagnostic biomarkers for patients with genuine sicca symptoms that do not meet current criteria, and prognostic biomarkers for patients at risk of lymphoma. Plasma extracellular vesicles (EVs) are promising biomarker candidates in several diseases, but their potential has not yet been explored in SjS. In this proof-of-concept study, we characterized EVs from primary SjS patients (pSS, n=12) at the phenotypic and proteomic levels, compared to EVs from healthy donor (HD, n=8) and systemic lupus erythematosus patients (SLE, n=12). Specific plasma EVs subpopulations, derived from neutrophils, endothelial, and epithelial cells, were found increased in pSS. We also identified a pSS proteomic signature in plasma EVs, including neutrophil-, epithelial-, and endothelial-related proteins, such as integrin alpha M (ITGAM), olfactomedin-4 (OLFM4), Ras-related protein RAB10, and CD36. Overall, our results support the relevance of plasma EVs as biomarkers in SjS.

## 1 Introduction

Sjögren syndrome (SjS) is a systemic autoimmune disease characterized by the destruction of exocrine glands epithelia, causing dryness of the mucosa (particularly oral, ocular and gynecological) called sicca symptoms. The incidence of SjS is approximately 3 to 11 per 100,000 individuals, and the female-to-male ratio vary across studies from 9:1 to 20:1 (1, 2). SjS can either be isolated, and is then referred to as primary SjS (pSS), or associated with other connective tissue diseases, such as systemic lupus erythematosus (SLE), systemic sclerosis, or rheumatoid arthritis, and is then termed secondary SjS (sSS). To date, diagnosis of SjS relies on an objective sicca syndrome associated with the positivity of at least one of the following criteria: i) anti-SSA/Ro60 autoantibodies, ii) lymphocytic infiltration of salivary glands. The treatment of SjS is mainly symptomatic, as there is no effective curative therapy (3). In addition to sicca symptoms, systemic disorders (including arthralgia, renal, pulmonary, and cardiovascular manifestations) can affect about half of the patients, but the main complication of SjS is lymphoma, which occurs in 5% of patients (1).

While the prevalence of anti-SSA60 autoantibodies is high in SjS (around 70%), some patients with positive histology are seronegative (1). Furthermore, salivary gland biopsy requires a trained operator and its interpretation can be challenging (4). There are also genuine sicca symptoms in patients who do not meet the diagnostic criteria for SjS. In addition, even if some prognostic factors of lymphoma have been identified, they are neither specific nor sufficient to dispense with careful follow-up of SjS patients (5). The identification of new, reliable, and minimally invasive diagnostic and prognostic biomarkers is therefore an important issue in SjS.

Extracellular vesicles (EVs) have recently emerged as potential biomarkers in various clinical settings, including cancer and auto-immune diseases (6). EVs are heterogeneous particles of 30 to 1000 nm in diameter composed of a lipid bilayer and containing a variety of biological materials, including proteins, lipids, and nucleic acids, which can mediate diverse biological effects. They are present in all tissues and biological fluids and their composition depends, notably, on their cell of origin (7). Salivary gland epithelial cells (SGEC) can release EVs containing SSA60 and SSB autoantigens, suggesting that EVs may be involved in the auto-immune response in SjS (8). pSS salivary and lacrimal EVs display specific protein and nucleic acid profiles, and have thus been proposed as potential biomarkers of the disease (9–13). However, few data on plasma EVs in pSS are available even if an increase in EV concentration, particularly of leucocyte and endothelial origins, has been reported, the latter being correlating with disease duration (14, 15).

Since plasma EVs fulfill all the criteria of promising biomarkers, we explored their concentration, distribution, cell origin and proteomic composition in pSS patients, compared to healthy donors (HD) and SLE patients.

## 2 Material and methods

### 2.1 Patients and samples

The research protocol was conducted under French legal guidelines and was approved by the Ethics Committee of Rennes Hospital (notice n° 21.139). Blood samples were obtained from 12 pSS patients, 12 SLE patients, and 8 HD. Inclusion criteria were: i) age > 18 years old; ii) for pSS patients, a pSS diagnosis proven by positivity of salivary gland biopsy and/or presence of anti-SSA/Ro60 antibodies; iii) for SLE patients, a SLE diagnosis in the absence of sSS (i.e. no sicca symptoms nor anti-SSA/Ro60 antibodies).

In brief, venous blood plasma was collected in citrate tubes, and within one hour following collection, citrate plasma was centrifuged twice at 2,500 g for 15 min to remove cells and cellular debris. The obtained platelet-free plasma (PFP) was stored at -80°C.

### 2.2 Flow cytometry

For flow cytometry, PFP were thawed and centrifuged at 14,000g for 2min to remove potential remaining cellular debris. Staining was performed on 10 µL of PFP for 15min at room temperature with FITC-AnnexinV (BD Biosciences, reference 556419) and various antibodies previously centrifuged at 14,000 g for 2min (**Table S1**). Stained EVs were then diluted and incubated for 30 to 180 min in 10 mM Hepes, 140 mM NaCl, 2.5 mM CaCl_2_, and 0.1% hirudin. To assess EV concentrations, MP-Count Beads (Biocytex) were added prior to acquisition on a CytoFLEX (Beckman Coulter) equipped with a violet side-scatter detector. EVs were defined as AnnexinV^pos^ events in a 100 to 900 nm size region determined by a mix of Megamix-Plus FSC and Megamix-Plus SSC (Biocytex). Flow cytometry data were analyzed with Kaluza Analysis software (Beckman Coulter).

### 2.3 Extracellular vesicles purification

All experiments related to EV purification and characterization were performed according to the minimal information for the studies of extracellular vesicles (MISEV) guideline from the International Society for Extracellular Vesicles (ISEV) (16). EVs were enriched using size exclusion chromatography (SEC). Briefly, 500 µL of PFP were thawed and loaded on a 10 mL Sepharose CL2-B column (GE Healthcare Life Sciences) before addition of phosphate-buffered saline solution (PBS). Fractions corresponding to the 4^th^, 5^th^, and 6^th^ mL (containing EVs) were collected as previously described (17). EVs purified in PBS were stored at -80°c, either unconcentrated or concentrated by ultracentrifugation at 100,000 g for 80 min on an Avanti J-30i centrifuge (Beckman Coulter).

### 2.4 Nanoparticle tracking analysis

Unconcentrated EVs were used for nanoparticle tracking analysis (NTA). After thawing, EVs were further diluted at a ratio of 1/50 or 1/100 in PBS. EV concentrations were assessed on a NanoSight NS300 (Malvern Panalytical), with a camera level of 30, and a detection threshold set to 5, under controlled temperature (25°C) and syringe pump speed. A minimum of 3 valid acquisitions of 60 seconds were used to assess the concentrations, determined as the mean of the different acquisitions.

### 2.5 Proteomic analysis

#### 2.5.1 Mass spectrometry analysis

*Sample Preparation:* For proteomic analysis, EVs were lysed in RIPA buffer (Thermo Scientific) before protein quantification by Bradford assay (Thermo Scientific), and protein concentrations were standardized across samples before storage at -80°C. Then, 5 µg of each sample was dried and solubilized in 10 µL 8M urea, 200 mM ammonium bicarbonate and then reduced in 5 mM dithiothreitol, pH 8 with vortexing at 37°C for 1 h. After cooling to room temperature, cysteines were alkylated by adding 10 mM iodoacetamide for 30 min in the dark. After diluting to 1 M urea with 100 mM ammonium bicarbonate pH 8.0, samples were digested with 0.2 µg trypsine/LysC (Promega) overnight, with vortexing at 37°C. Samples were then loaded onto homemade C18 StageTips packed by stacking three AttractSPE Disk (#SPE-Disk-Bio-C18, Affinisep) for desalting. Peptides were eluted using 40/60 MeCN/H2O + 0.1% formic acid, vacuum concentrated to dryness and reconstituted in 10 µl injection buffer (0.3% TFA) before liquid chromatography-tandem mass spectrometry (LC-MS/MS) analysis.

*LC-MS/MS Analysis:* Online chromatography was performed with an RSLCnano system (Ultimate 3000, Thermo Scientific) coupled to an Orbitrap Exploris 480 mass spectrometer (Thermo Scientific). Peptides were first trapped on a C18 column (75 μm inner diameter × 2 cm; nanoViper Acclaim PepMap™ 100, Thermo Scientific) with buffer A (2/98 MeCN/H2O in 0.1% formic acid) at a flow rate of 2.5 µL/min over 4 min. Separation was then performed on a 50 cm x 75 μm C18 column (nanoViper Acclaim PepMap™ RSLC, 2 μm, 100 Å, Thermo Scientific) regulated to a temperature of 50°C with a linear gradient of 2% to 25% buffer B (100% MeCN in 0.1% formic acid) at a flow rate of 300 nL/min over 91 min. MS full scans were performed in the ultrahigh-field Orbitrap mass analyzer in ranges *m*/*z* 375–1500 with a resolution of 120,000 at *m*/*z* 200. The top 20 most intense ions were isolated and subjected to further fragmentation *via* high-energy collision dissociation (HCD) activation and acquired at resolution of 15,000 with the auto gain control (AGC) target set to 100%. We selected ions with charge state from 2+ to 6+ for screening.

Normalized collision energy (NCE) was set at 30% and the dynamic exclusion at 40s.

#### 2.5.2 Data processing protocol

For identification, the data was searched against the Homo Sapiens (UP000005640) UniProt database using Sequest-HT through proteome discoverer (version 2.4). Enzyme specificity was set to trypsin and a maximum of two missed cleavages sites were allowed. Oxidized methionine, Met-loss, Met-loss-Acetyl and N-terminal acetylation were set as variable modifications. Carbamidomethylation of cysteins were set as fixed modification. Maximum allowed mass deviation was set to 10 ppm for monoisotopic precursor ions and 0.02 Da for MS/MS peaks. The resulting files were further processed using myProMS v3.9.3 [(18); https://github.com/bioinfo-pf-curie/myproms]. FDR calculation used Percolator (19) and was set to 1% at the peptide level for the whole study. The label-free quantification was performed by peptide Extracted Ion Chromatograms (XICs), reextracted across all conditions and computed with MassChroQ version 2.2.21 (20).

For protein quantification, XICs from proteotypic peptides shared between compared conditions (TopN matching) and missed cleavages were allowed. Median and scale normalization was applied on the total signal to correct the XICs for each biological replicate for total signal and global variance biases. To estimate the significance of the change in protein abundance, a linear model (adjusted on peptides and biological replicates) was performed, and p-values were adjusted using the Benjamini–Hochberg false discovery rate (FDR) procedure. Protein with at least two total peptides across three biological replicates of an experimental condition, a two-fold enrichment and an adjusted p-value ≤ 0.05 were considered significantly enriched in sample comparison. Proteins unique to a condition were also considered if they matched the peptide criteria. Label-free quantification (LFQ) was also performed following the algorithm as described (21) with the minimum number of peptide ratios set to 2 peptides and with the large ratios stabilization feature. Also, for all conditions (SLE, pSS & HD), protein molar and mass percentage were estimated by using Top 3 (22) (including proteins with less than 3 peptides) or iBAQ (23) as the Protein Quantification Index and the direct proportionality model (24).

### 2.6 Statistical analysis

Statistical analyses were performed and figures were generated with Graphpad Prism 9.1.4 and R v4.0.0, using Rstudio v1.4.1717. Principal component analysis (PCA) was performed with the FactoMineR package (v2.4) on log10-transformed LFQ data, on all proteins (n=248) identified with ≥ 2 peptides, with 34% missing values allowed per protein. Missing values were imputed using the missMDA package (v1.18). Volcano plots were obtained using the EnhancedVolcano package v1.8.0.

## 3 Results

### 3.1 Patients

Analyses were performed on plasma samples from 12 pSS patients, 12 SLE patients, and 8 sex- and age-matched HD, whose characteristics are detailed in **Table 1**. All pSS patients meet ACR/EULAR 2016 classification criteria and present anti-SSA/Ro60 antibodies, whereas all SLE patients were negative for anti-SSA/Ro60 antibodies and did not report sicca symptoms. Nine out the 12 pSS patients present non-threating systemic manifestations such as articular, cutaneous, pulmonary, or cardio-vascular involvement.

**Table 1:** Characteristics of patients and controls at sampling time.

	HD	SLE	pSS
Demography			
n	8	12	12
Age in years (mean ± SD)	43 ± 13	41 ± 11	50 ± 17
Women (%)	7 (88%)	12 (100%)	11 (92%)
Clinical features			
Disease duration in years (mean ± SD)	–	7.5 ± 8	11.3 ± 9
Sicca symptoms (%)	–	0 (0%)	12 (100%)
SjS systemic manifestations^#^ (%)	–	–	9 (75%)
SLEDAI/ESSDAI (mean ± SD)	–	8.7 ± 8.5	5.6 ± 10
Laboratory results			
Positive salivary gland biopsy^##^ (%)	–	–	7 (58%)
Anti-nuclear antibodies (%) (≥1/160)	–	11 (92%)	11 (92%)
Anti-ADN antibodies (%) (>15 UI/mL)	–	10 (83%)	0 (0%)
Anti-SSA/Ro60 antibodies (%)	–	0 (0%)	12 (100%)
Anti-SSB/La antibodies (%)	–	0 (0%)	4 (33%)
Anti-Sm antibodies (%)	–	5 (42%)	0 (0%)
Anti-RNP antibodies (%)	–	6 (50%)	0 (0%)
Low complement activity (%) (<35 U/mL)	–	2 (17%)	0 (100%)
Low complement C3 (%) (<0.85 g/L)	–	1 (8%)	0 (0%)
Low complement C4 (%) (<0.08 g/L)	–	2 (17%)	0 (0%)
Treatment			
DMTs^###^ (%)	–	10 (83%)	5 (42%)

HD, healthy donors; SLE, systemic lupus erythematosus; pSS, primary Sjögren syndrome; SjS, Sjögren syndrome; SD, standard deviation; SLEDAI, Systemic Lupus Erythematosus Disease Activity Index; ESSDAI, EULAR Sjogren’s Syndrome Disease activity Index; DMTs, disease modifying therapies. ^#^ includes articular, cutaneous, pulmonary, and cardio-vascular involvement; ^##^ focal lymphocytic sialoadenitis defined as a focus score > 1; ^###^ includes hydroxychloroquine, methotrexate, azathioprine, mycophenolate mofetil, corticosteroids.

### 3.2 EV concentrations vary between patients and controls

**Figure 1:**
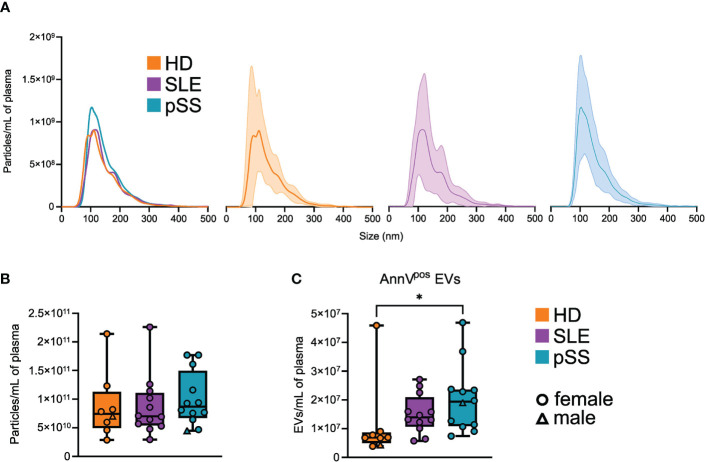
Quantitative profile of plasma extracellular vesicles (EVs) in primary Sjogren syndrome. **(A)** Average size distribution of plasma EVs isolated by size exclusion chromatography (SEC), in primary Sjogren syndrom (pSS, n=12), systemic lupus erythematosus (SLE, n=12), and healthy donors (HD, n=8), assessed by nanoparticle analysis (NTA). Error range indicates ± 1 standard error (SD) of the mean. **(B)** Plasma EVs concentration in pSS (n=12), SLE (n=12), and HD (n=8) assessed by NTA after SEC isolation. Whiskerboxes indicate median, 25^th^ and 75^th^ percentiles, minimum and maximum values. **(C)** Plasma AnnexinV^pos^ (AnnV) EVs concentration in primary pSS (n=12), SLE (n=12), and HD (n=8) assessed by flow cytometry. Kruskal-Wallis, multiple comparison with Dunn correction, *p<0.05.

We first sought to assess the quantitative profiles of plasma EVs in pSS patients (n=12) compared to controls (SLE, n=12 and HD, n=8). We used two distinct approaches: NTA on SEC purified EVs and flow cytometry on whole PFP. Post-SEC NTA allowed us to determine the size distribution and concentration of plasma particles, while limiting the bias of circulating lipoproteins. We observe a similar size distribution of plasma particles among pSS patients, SLE patients, and HD, with a peak around 110 nm and a valley around 150 nm, separating exosomes from microvesicles (**Figure 1A**). No statistical difference was found in the particle concentrations between the three groups (**Figure 1B**). Using flow cytometry, we focused on AnnexinV^pos^ EVs, which display phosphatidylserine on their surface. This peculiar EV compartment was significantly more abundant in pSS and SLE patients than in HD (**Figure 1C**). Overall, plasma EV concentrations appear to differ between pSS patients and HD, a difference which may be based on specific subpopulations of EVs.

### 3.3 The abundance of specific EV subpopulations is altered in pSS

**Figure 2:**
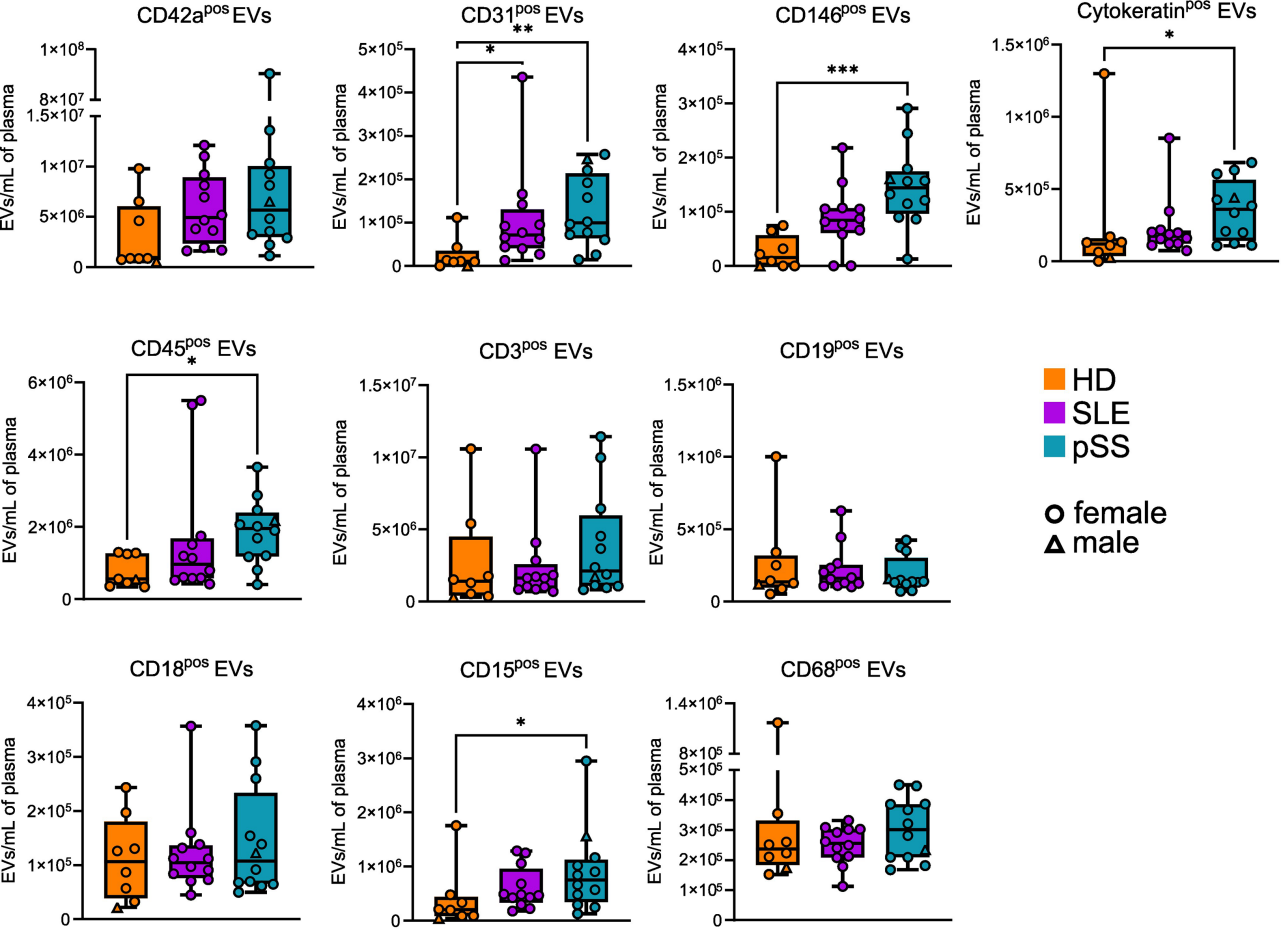
Phenotypic profile of plasma extracellular vesicles (EVs) in primary Sjogren syndrome. Plasma concentration of EVs displaying different markers in primary Sjogren syndrom (pSS, n=12), systemic lupus erythematosus (SLE, n=12), and healthy donors (HD, n=8). Kruskal-Wallis, multiple comparison with Dunn correction, *p<0.05, **p<0.01, ***p<0.001. Whiskerboxes indicate median, 25^th^ and 75^th^ percentiles, minimum and maximum values.

To refine our quantitative analysis of EVs subpopulations and explore their cell of origin, we assessed the expression of non-hematopoietic and hematopoietic markers on the plasma AnnexinV^pos^ EVs by flow cytometry in pSS (n=12), SLE (n=12), and HD (n=8). Overall, numerous EV subsets were statistically increased in pSS (and less markedly in SLE) compared to HD (**Figure 2**). EVs expressing the platelet marker CD42a were, as expected, the most abundant population, and no difference was found between pSS, SLE, and HD samples. By contrast, CD31^pos^ and CD146^pos^ endothelium-derived EVs were found to be significantly more abundant in pSS compared to HD, possibly reflecting endothelial activation and/or damage in this condition. Of note, an increase in CD31^pos^ endothelium-derived EVs was also found in SLE, where endothelial activation and/or damage may also occur. In addition, the concentration of cytokeratin^pos^ EVs was significantly higher in pSS patients, which could reflect epithelial damage. Regarding the hematopoietic lineage, CD45^pos^ leukocyte-derived EVs were significantly elevated in pSS, but not in SLE, compared to HD. More specifically, neutrophil-derived CD15^pos^, but not T cell-derived CD3^pos^, B cell-derived CD19^pos^, nor myeloid-derived CD18^pos^ and CD68^pos^ EVs were increased in pSS. Of note, CD15 was recently proposed as the most sensitive and specific marker of neutrophil-derived EVs, as opposed to CD18, which is present on both monocyte- and neutrophil-derived EVs (25). Taken together, our FC results confirm that specific EVs subpopulations, particularly those derived from endothelial cells, epithelial cells, and neutrophils, are more abundant in pSS.

### 3.4 Proteomic analysis of plasma EVs uncovers potential pSS biomarkers

**Figure 3:**
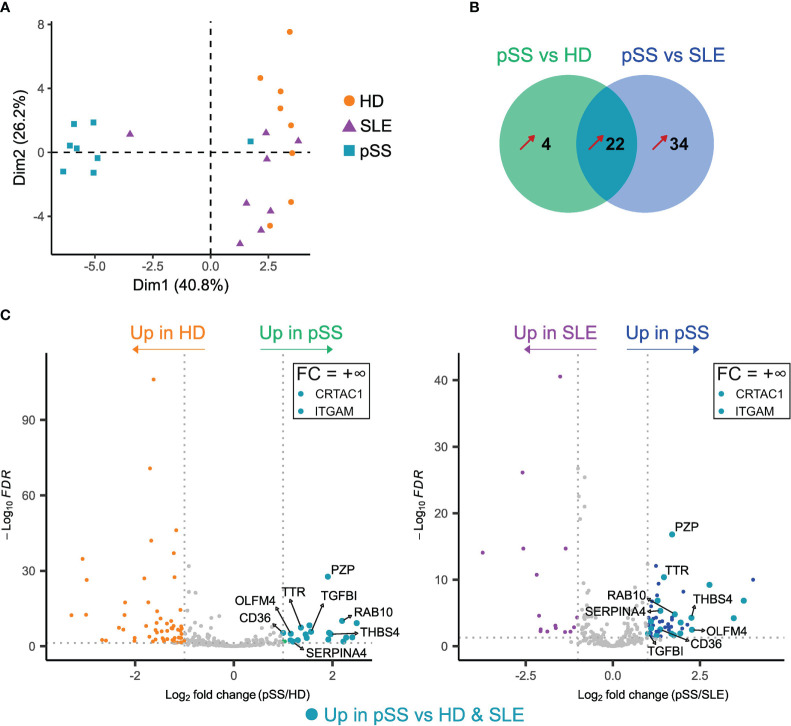
Proteomic analysis of plasma extracellular vesicles (EVs) in primary Sjogren syndrome (pSS) identifies potential pSS biomarkers. Label-free quantification of plasma EVs was performed on pSS patients (n=8), systemic lupus erythematosus (SLE) patients (n=8) and healthy donors (n=8). **(A)** Principal component analysis (PCA) of all proteins (n=248) identified with ≥ 2 peptides in at least 2/3 of the analyzed samples. **(B)** Venn diagram representing the number of proteins upregulated in pSS *versus* HD and in pSS *versus* SLE. Fold-change (FC) > 2, adjusted p-value (Padj) < 0.05. Only proteins identified with ≥ 2 peptides and in at least 3 samples from one of the groups are represented. Unique proteins (+∞ or -∞) to only one group were also considered. **(C)** Volcano plots of differentially expressed proteins between pSS and HD (left) and pSS and SLE (right). Only proteins identified with ≥ 2 peptides and in at least 3 samples from one of the groups are shown. Proteins with a FC = +∞ or -∞ are not shown, unless found upregulated in pSS versus both HD and SLE (see **Tables S2**, **S3**). Proteins found upregulated in pSS *versus* both HD and SLE are highlighted by larger turquoise points.

Having explored EV putative origin, we focused on the protein content of EVs in pSS. We performed a quantitative proteomic analysis of EVs from 8 samples of each group (pSS, SLE, and HD). We identified a mean of 752 proteins with a standard deviation (SD) of 56 in the pSS group, a mean of 791 proteins with a SD of 47 in the SLE group, and a mean of 908 proteins with a SD of 83 in the HD group. Unsupervised exploration by PCA showed that the different samples could be globally grouped according to their population of origin (**Figure 3A**). Of note, one SLE sample seem to cluster with the pSS samples, while one pSS patient is closer to SLE and HD samples than other pSS. We did not identify any clinical or biological data explaining this discrepancy. Even if these 2 patients did not display at the time of sampling any confounding clinical feature, we cannot exclude that a prolonged follow-up would reveal a clinical evolution towards either SLE or Sjögren syndrome.

To identify a specific pSS signature, we performed differentially expressed proteins (DEP) analyses between pSS and either HD and SLE patients (**Tables S2**, **S3**), followed by a Venn diagram highlighting proteins upregulated in pSS compared to the 2 other conditions with a fold change (FC) > 2 and an adjusted p-value ≤ 0.05 (**Figure 3B**). Of the 22 proteins included in this pSS-specific protein signature, 12 were immunoglobulin (Ig) proteins, possibly reflecting B-cell activation and associated skewing of the B cell receptor (BCR) repertoire in pSS (26, 27). Other secreted plasma proteins included transthyretin (TTR), thrombospondin-4 (THBS4), kallistatin (SERPINA4), cartilage acidic protein 1 (CRTAC1), as well as the transforming growth factor beta-induced protein (TGFBI) (**Figure 3C**). We also found a significant upregulation of the pregnancy zone protein (PZP), which is usually elevated in pregnancy and inflammation, and which could play roles in immunoregulation (28). The RAS-related protein RAB10 was present in the pSS signature. RAB10 might be involved in exosome biogenesis (7), and is known to play a role in polarized transport in epithelial cells (29). Its upregulation in pSS may thus be linked to epithelial damage, in association with the increased levels of epithelium-derived EVs shown by flow cytometry. The scavenger receptor CD36 was also found to be upregulated in pSS. CD36 can be expressed by many cell subsets including endothelial cells (30); its upregulation in pSS EVs may then be associated to the increased levels of endothelium-derived EVs and reflect a specific state of activation of the endothelial compartment in this disease. Another DEP was olfactomedin-4 (OLFM4), a glycoprotein expressed by immature myeloid cells and mature neutrophils, which has been proposed as a biomarker in several autoimmune diseases and cancers (31). Finally, the upregulation of the myeloid marker integrin alpha M (ITGAM)/CD11b in pSS EVs may, like OLFM4, be related to the increased concentration of neutrophil-derived EVs found using flow cytometry and reflect a pSS-specific activation of these cells. Altogether, pSS plasma EVs display a specific proteome, which could allow identification of new biomarkers of this disease.

## 4 Discussion

Identification of new specific and non-invasive biomarkers is a key challenge in SjS. Herein, we provide evidence that plasma EVs are relevant biomarker candidates in a proof-of-concept study on 3 small groups of pSS patients, SLE patients, and HD. We show that plasma EVs of pSS patients display specific profiles compared to HD, as well as to SLE (another connective tissue disease). We propose for the first time a proteomic analysis of plasma EVs of pSS patients, revealing new potential plasma pSS biomarkers such as OLFM4 and RAB10.

Using flow cytometry, we could observe higher levels of EVs in pSS plasma (and a similar tendency, although not significant, in SLE) compared to HD, in line with previous published data (15).

Conversely, no statistical difference was found between groups with NTA. EVs levels assessed by FC were also significantly lower than the concentration of plasma particles measured by NTA. These discrepancies are consistent with the literature and might be explained by several factors (32), some of which were inherent in the design of this study. First, flow cytometry was performed on whole plasma focusing on AnnexinV^pos^ EVs, whereas NTA was performed on SEC purified particles. SEC can therefore introduce a selective bias in purified particles. Nevertheless, we considered this step necessary to optimize EV enrichment and prevent lipoprotein contamination (17, 33). The AnnexinV staining may also introduce a bias towards EVs with a PS-enriched membrane. Second, samples from pSS patients seem more heterogeneous than those from HD and SLE, which might contribute to explain the lack of statistical difference between EV concentrations in pSS patients and controls using NTA. Actually, pSS EVs show a trend towards increased concentration compared to SLE and HD with NTA, at least in a subgroup of patients. The same tendency can be observed in flow cytometry, in pSS as in SLE, with a significant inter-sample heterogeneity suggesting distinct subgroups. Larger cohorts are needed to assess this heterogeneity and potentially delineate patient subgroups, which may differ for the EVs levels, in association with disease activity. Altogether, the plasma EV compartment size and distribution are altered in pSS.

In line with the scant literature on this topic, we found an increase in endothelial-derived plasma EVs in pSS (14), probably reflecting endothelial activation. Conversely, no statistical differences was found for for platelet-derived EVs, in contrast with previous published data (15), perhaps due to the small size of our groups. Nevertheless, the fact that this largely predominant EV population does not explain the differences in AnnV^pos^ EVs concentrations between pSS and SLE patients and HD emphasizes the differences identified in less represented EV populations. Among these, epithelium-derived EVs were more abundant in pSS than HD. These EVs could be released by affected salivary or ocular epithelium. It has indeed been shown that SGEC are prone to release cytokeratin^pos^ EVs (8). Consistent with the literature, we found an increase in leukocyte-derived EVs in pSS, and not SLE, compared to HD (15), which could reflect a specific immune cell activation. Refining this analysis with different markers, we identified for the first time alterations in the concentrations of lineage-specific EVs in pSS. While T and B cells are the most studied immune cell subsets in this disease, B/T-derived EVs were not increased in pSS compared to HD. The small number of B cell-derived EVs could however be due to low sensitivity of the CD19 staining. Interestingly, neutrophil-dervied EVs were increased in pSS compared to HD. This highlights the potential crucial implication of the myeloid lineage in pSS pathogenesis, although this population is poorly explored in this pathology (5, 34).

The involvement of the myeloid lineage, as well as innate immunity, in pSS is supported by our proteomic data on plasma EVs. Among upregulated proteins in pSS EVs compared to controls, we found, notably, CD11b and OLFM4. CD11b is largely expressed by neutrophils, and was previously found upregulated at the protein level in whole saliva from pSS patients compared to HD (35). CD11b is also enriched in whole saliva from head and neck cancer compared to pSS patients and may be of interest as a biomarker (36). OLFM4 is produced by neutrophils and has been described as a biomarker of inflammation in auto-immune diseases and in cancer, notably in whole saliva from head and neck cancer compared to pSS patients (31, 36). Its potential as a diagnostic and/or prognostic biomarker in pSS would deserve further exploration. Overall, despite a clear activation of the myeloid compartment in pSS (37), the precise role as pathogenic or bystander cells of neutrophils and neutrophil-derived EVs in this pathology remains to be explored. Intriguingly, we could identify in EVs from pSS patients one protein possibly related to epithelial damage, in line with the increased number of epithelial-derived EVs noted by flow cytometry. RAB10, which is involved in intracellular trafficking in epithelial cells (29), is specifically enriched in plasma EVs from pSS patients compared to SLE patients and HD and may reflect the specific state of activation of damaged epithelium in pSS. Whether EV-derived RAB10 could be considered as a non-invasive diagnostic marker of pSS requires further validation on a larger cohort of patients, including pSS patients seronegative for anti-SSA60 antibodies.

Our study has several limitations. First, our study groups are quite small, which could prevent the identification of some differences between populations as well as the assessment of inter-patient heterogeneity. Second, we cannot exclude that our AnnexinV-based identification of plasma EVs by FC may be confounded by lipoproteins, which also bind AnnexinV, as recently highlighted by Botha et al. (38). Lastly, our proteomic data raise the question of potential plasma contamination, which would be expected in this kind of approach. Still, it is quite impossible to differentiate real plasma proteins from proteins bound to the EV membrane. However, we optimized EV isolation using SEC, which has been shown to drastically reduce contamination by plasma proteins, without compromising EV yield, compared to ultracentrifugation alone (17, 32, 33).

In conclusion, this proof-of-concept study emphasizes the potential of plasma EVs as pSS biomarkers. The validation of diagnostic and prognostic biomarkers would require larger prospective cohorts, including seronegative patients, and long-term follow-up to assess disease complications and particularly the risk of lymphoma.

## Data availability statement

The datasets presented in this study can be found in online repositories. This data can be found at https://www.ebi.ac.uk/pride/archive/projects/PXD041452.

## Ethics statement

The research protocol involving human participants was conducted under French legal guidelines and was approved by the Ethics Committee of Rennes Hospital (notice n° 21.139).

## Author contributions

JF designed research, performed experiments, analyzed data, and wrote the manuscript; AP performed experiments; FZ curated and analyzed clinical data; FD carried out the MS experimental work; PP analyzed MS data; DL supervised MS and data analysis; KT supervised research and provided funding; ED designed and supervised research and wrote the manuscript. All authors contributed to the article and approved the submitted version.
