# Supplementary material for: Phenotypic and proteomic analysis of plasma extracellular vesicles highlights them as potential biomarkers of primary Sjögren syndrome
Source: Front Immunol. 2023 Jul 17;14:1207545. doi: 10.3389/fimmu.2023.1207545 (PMC10388367; doi:10.3389/fimmu.2023.1207545)
Supplement: Supplementary file 1 [file Table_1.docx]

Table S1 | **Flow cytometry antibodies**

| **Target** | **Fluorochrome** | **Clone** | **Reference** | **Manufacturer** |
| --- | --- | --- | --- | --- |
| CD146 | PE | 541-10B2 | 130-092-853 | Miltenyi Biotec |
| CD31 | APC | AC128 | 130-119-891 | Miltenyi Biotec |
| CD42a | PE-Vio770 | REA209 | 130-100-970 | Miltenyi Biotec |
| CD15 | PE | 80H5 | IM1954U | Beckman Coulter |
| CD18 | APC | 6.7 | 551060 | Beckton Dickinson |
| CD68 | PerCP-Cy5.5 | Y1/82A | 333814 | Biolegend |
| Pan Cytokeratin 14, 15, 16, 19 | A647 | KA4 | 563648 | Beckton Dickinson |
| CD3 | PE-Cy7 | SK7 | 557851 | Beckton Dickinson |
| CD45 | PerCP-Cy5.5 | HI30 | 564105 | Beckton Dickinson |
| CD19 | PerCP-Cy5.5 | SJ25C1 | 332780 | Beckton Dickinson |
