# Supplementary material for: Phenotypic and proteomic analysis of plasma extracellular vesicles highlights them as potential biomarkers of primary Sjögren syndrome
Source: Front Immunol. 2023 Jul 17;14:1207545. doi: 10.3389/fimmu.2023.1207545 (PMC10388367; doi:10.3389/fimmu.2023.1207545)
Supplement: Supplementary file 2 [file Table_2.docx]

Table S2 | **Differentially expressed proteins in pSS vs HD**

Differentially Expressed Proteins are defined by |FC| > 2 and Padj < 0.05. Only proteins identified with a minimum of 2 peptides and in at least 3 samples of one of the groups are shown. Proteins unique (+∞ or -∞ ) to a condition were also considered if they matched the peptide criteria. Common upregulated proteins with pSS vs SLE comparison are highlighted in red; immunoglobulin proteins are highlighted in grey. pSS, primary Sjogren syndrome; DEP, differentially expressed proteins; HD, healthy donors; FC, fold change; Padj, adjusted p-value.

| **Protein** | **Gene** | **Log2FC** | **Padj** |
| --- | --- | --- | --- |
| Q9NQ79 | CRTAC1 | +∞ | - |
| Q15833 | STXBP2 | +∞ | - |
| P11215 | ITGAM | +∞ | - |
| P01854 | IGHE | +∞ | - |
| P24043 | LAMA2 | +∞ | - |
| P01599 | IGKV1-17 | 2.49 | 6.13E-10 |
| P01700 | IGLV1-47 | 2.40 | 2.91E-04 |
| A0A075B6I1 | IGLV4-60 | 2.28 | 3.49E-04 |
| P80748 | IGLV3-21 | 2.22 | 1.32E-02 |
| P61026 | RAB10 | 2.20 | 7.96E-11 |
| P35443 | THBS4 | 1.96 | 1.61E-05 |
| P0DTE1 | IGHV3-38-3 | 1.93 | 4.52E-06 |
| A0A0G2JRQ6 | A0A0G2JRQ6 | 1.92 | 2.09E-03 |
| P20742 | PZP | 1.91 | 2.10E-28 |
| Q15582 | TGFBI | 1.57 | 1.66E-06 |
| P01857 | IGHG1 | 1.53 | 5.05E-09 |
| Q6UX06 | OLFM4 | 1.48 | 4.75E-04 |
| P01880 | IGHD | 1.46 | 1.93E-05 |
| P02766 | TTR | 1.36 | 3.64E-08 |
| P01619 | IGKV3-20 | 1.30 | 4.10E-03 |
| P29622 | SERPINA4 | 1.20 | 1.46E-02 |
| A0A0C4DH31 | IGHV1-18 | 1.16 | 1.06E-05 |
| A0A075B6H9 | IGLV4-69 | 1.15 | 4.65E-03 |
| O15400 | STX7 | 1.04 | 1.21E-02 |
| P07195 | LDHB | 1.01 | 1.28E-02 |
| P16671 | CD36 | 1.00 | 4.80E-06 |
| P01860 | IGHG3 | -1.00 | 5.88E-03 |
| P35527 | KRT9 | -1.05 | 8.71E-08 |
| P01859 | IGHG2 | -1.06 | 1.31E-05 |
| P01602 | IGKV1-5 | -1.06 | 2.84E-02 |
| P05155 | SERPING1 | -1.07 | 1.28E-08 |
| P51884 | LUM | -1.07 | 4.71E-04 |
| P18428 | LBP | -1.07 | 2.79E-02 |
| P55056 | APOC4 | -1.08 | 5.16E-04 |
| P02790 | HPX | -1.08 | 3.49E-15 |
| P02749 | APOH | -1.08 | 6.15E-07 |
| O95445 | APOM | -1.11 | 3.70E-04 |
| P63104 | YWHAZ | -1.13 | 2.63E-07 |
| P17936 | IGFBP3 | -1.14 | 3.24E-02 |
| P01023 | A2M | -1.17 | 7.44E-47 |
| P11277 | SPTB | -1.20 | 5.16E-04 |
| P02671 | FGA | -1.20 | 3.62E-28 |
| A0A0B4J1Y9 | ND4L | -1.21 | 1.15E-02 |
| P02768 | ALB | -1.22 | 9.33E-38 |
| P14618 | PKM | -1.22 | 8.54E-09 |
| P0C0L4 | C4A | -1.24 | 3.42E-03 |
| P26038 | MSN | -1.24 | 8.63E-08 |
| P06733 | ENO1 | -1.26 | 6.51E-08 |
| O75636 | FCN3 | -1.27 | 8.89E-11 |
| P13224 | GP1BB | -1.35 | 1.47E-03 |
| P20851 | C4BPB | -1.35 | 7.58E-07 |
| P01591 | JCHAIN | -1.35 | 9.56E-10 |
| P02538 | KRT6A | -1.41 | 1.28E-02 |
| P13987 | CD59 | -1.43 | 2.14E-03 |
| P01817 | IGHV2-5 | -1.44 | 6.21E-04 |
| B0I1T2 | MYO1G | -1.46 | 1.17E-06 |
| P35908 | KRT2 | -1.48 | 9.41E-12 |
| P68871 | HBB | -1.55 | 1.21E-14 |
| P02549 | SPTA1 | -1.56 | 3.52E-06 |
| P27918 | CFP | -1.56 | 5.24E-10 |
| A0A075B6I0 | IGLV8-61 | -1.59 | 2.03E-03 |
| P02730 | SLC4A1 | -1.59 | 3.70E-07 |
| P04275 | VWF | -1.63 | 8.06E-107 |
| P01714 | IGLV3-1 | -1.64 | 1.35E-02 |
| P02647 | APOA1 | -1.67 | 9.89E-43 |
| P02787 | TF | -1.70 | 1.83E-71 |
| P61769 | B2M | -1.71 | 1.58E-04 |
| O43866 | CD5L | -1.73 | 3.44E-18 |
| P02654 | APOC1 | -1.78 | 8.93E-09 |
| P02649 | APOE | -1.81 | 1.04E-27 |
| A0A075B7B8 | IGHV3OR16-12 | -2.01 | 4.72E-04 |
| Q8WUA8 | TSKU | -2.01 | 7.29E-03 |
| P69905 | HBA1 | -2.20 | 3.85E-18 |
| P05090 | APOD | -2.22 | 3.80E-13 |
| P01861 | IGHG4 | -2.24 | 2.37E-07 |
| P21926 | CD9 | -2.33 | 4.43E-08 |
| P01780 | IGHV3-7 | -2.59 | 5.27E-03 |
| P01705 | IGLV2-23 | -2.67 | 3.21E-03 |
| P02745 | C1QA | -2.98 | 4.22E-27 |
| P35542 | SAA4 | -2.99 | 3.00E-13 |
| P02652 | APOA2 | -3.07 | 1.83E-35 |
| P02655 | APOC2 | -3.29 | 4.55E-13 |
| P11166 | SLC2A1 | -∞ | - |
| O00560 | SDCBP | -∞ | - |
| P02042 | HBD | -∞ | - |
| Q86YZ3 | HRNR | -∞ | - |
| P11597 | CETP | -∞ | - |
| O00592 | PODX | -∞ | - |
| P05452 | CLEC3B | -∞ | - |
| P15311 | EZR | -∞ | - |
| P31146 | CORO1A | -∞ | - |
| P00915 | CA1 | -∞ | - |
| P80723 | BASP1 | -∞ | - |
| P0DP25 | CALM3 | -∞ | - |
| P14151 | SELL | -∞ | - |
| O75955 | FLOT1 | -∞ | - |
| Q9Y624 | F11R | -∞ | - |
| Q96KN2 | CNDP1 | -∞ | - |
| Q9NZP8 | C1RL | -∞ | - |
| Q9Y6Z7 | COLEC10 | -∞ | - |
| Q9H4G4 | GLIPR2 | -∞ | - |
| Q96IY4 | CPB2 | -∞ | - |
| Q15599 | SLC9A3R2 | -∞ | - |
| P63218 | GNG5 | -∞ | - |
| P07355 | ANXA2 | -∞ | - |
| P52566 | ARHGDIB | -∞ | - |
| P00558 | PGK1 | -∞ | - |
| P04083 | ANXA1 | -∞ | - |
| P62879 | GNB2 | -∞ | - |
| Q06830 | PRDX1 | -∞ | - |
| P04180 | LCAT | -∞ | - |
| P31946 | YWHAB | -∞ | - |
| Q658P3 | STEAP3 | -∞ | - |
| P61225 | RAP2B | -∞ | - |
| P08754 | GNAI3 | -∞ | - |
| Q9Y696 | CLIC4 | -∞ | - |
