# Supplementary material for: Phenotypic and proteomic analysis of plasma extracellular vesicles highlights them as potential biomarkers of primary Sjögren syndrome
Source: Front Immunol. 2023 Jul 17;14:1207545. doi: 10.3389/fimmu.2023.1207545 (PMC10388367; doi:10.3389/fimmu.2023.1207545)
Supplement: Supplementary file 3 [file Table_3.docx]

Table S3 | **Differentially expressed proteins in pSS vs SLE**

Differentially Expressed Proteins are defined by |FC| > 2 and Padj < 0.05. Only proteins identified with a minimum of 2 peptides and in at least 3 samples of one of the groups are shown. Proteins unique (+∞ or -∞ ) to a condition were also considered if they matched the peptide criteria. Common upregulated proteins with pSS vs HD comparison are highlighted in red, immunoglobulin proteins are highlighted in grey. pSS, primary Sjogren syndrome; DEP, differentially expressed proteins; SLE, systemic lupus erythematosus; FC, fold change; Padj, adjusted p-value.

| **Protein** | **Gene** | **Log2FC** | **Padj** |
| --- | --- | --- | --- |
| Q96IY4 | CPB2 | +∞ | - |
| Q9NQ79 | CRTAC1 | +∞ | - |
| P11215 | ITGAM | +∞ | - |
| Q13093 | PLA2G7 | +∞ | - |
| P01854 | IGHE | +∞ | - |
| P05362 | ICAM1 | +∞ | - |
| P02776 | CXCL4 | +∞ | - |
| A6NGR9 | MROH6 | 4.03 | 9.95E-11 |
| P0DTE1 | IGHV3-38-3 | 3.76 | 1.41E-07 |
| P01700 | IGLV1-47 | 3.47 | 5.97E-05 |
| P01599 | IGKV1-17 | 2.77 | 5.85E-10 |
| Q6UX06 | OLFM4 | 2.27 | 3.29E-03 |
| P35443 | THBS4 | 2.26 | 5.19E-05 |
| P02775 | PPBP | 2.14 | 5.77E-04 |
| P01703 | IGLV1-40 | 2.02 | 6.22E-09 |
| A0A0J9YX35 | IGHV3-64D | 1.99 | 2.14E-03 |
| P01619 | IGKV3-20 | 1.95 | 2.72E-04 |
| P80748 | IGLV3-21 | 1.94 | 1.19E-02 |
| P61026 | RAB10 | 1.78 | 1.59E-05 |
| A0A0G2JRQ6 | A0A0G2JRQ6 | 1.75 | 2.19E-02 |
| P20742 | PZP | 1.70 | 1.54E-17 |
| O15031 | PLXNB2 | 1.70 | 4.77E-05 |
| A0A075B6I1 | IGLV4-60 | 1.68 | 1.28E-02 |
| P17858 | PFKL | 1.68 | 1.19E-03 |
| P04430 | IGKV1-16 | 1.61 | 3.42E-03 |
| P02656 | APOC3 | 1.59 | 1.11E-03 |
| P26927 | MST1 | 1.59 | 4.72E-04 |
| P08185 | SERPINA6 | 1.50 | 1.42E-03 |
| P02766 | TTR | 1.47 | 4.14E-11 |
| O14745 | SLC9A3R1 | 1.38 | 3.18E-02 |
| A0A0C4DH31 | IGHV1-18 | 1.36 | 4.69E-06 |
| P29622 | SERPINA4 | 1.36 | 2.98E-03 |
| P01764 | IGHV3-23 | 1.35 | 2.09E-04 |
| P01601 | IGKV1D-16 | 1.32 | 4.67E-03 |
| A0A0B4J1V6 | ND4L | 1.32 | 6.64E-03 |
| P24821 | TNC | 1.31 | 4.02E-10 |
| P01857 | IGHG1 | 1.29 | 1.39E-07 |
| P19652 | ORM2 | 1.27 | 2.20E-08 |
| A0A075B6H9 | IGLV4-69 | 1.25 | 2.35E-02 |
| P60660 | MYL6 | 1.25 | 6.23E-03 |
| P01877 | IGHA2 | 1.24 | 4.97E-05 |
| P68871 | HBB | 1.24 | 8.29E-13 |
| P16070 | CD44 | 1.17 | 7.16E-05 |
| P04406 | GAPDH | 1.16 | 7.34E-04 |
| Q96Q89 | KIF20B | 1.13 | 4.58E-02 |
| P00748 | F12 | 1.12 | 1.75E-07 |
| P01880 | IGHD | 1.10 | 2.04E-03 |
| Q15485 | FCN2 | 1.10 | 9.92E-07 |
| P16671 | CD36 | 1.09 | 1.91E-04 |
| Q13790 | APOF | 1.09 | 1.07E-02 |
| Q9BYG3 | NIFK | 1.08 | 1.84E-02 |
| P00739 | HPR | 1.06 | 8.63E-03 |
| P01834 | IGKC | 1.03 | 5.22E-05 |
| P04432 | IGKV1D-39 | 1.03 | 6.51E-04 |
| P09172 | DBH | 1.02 | 1.19E-02 |
| Q15582 | TGFBI | 1.01 | 1.30E-02 |
| P04004 | VTN | -1.02 | 4.77E-05 |
| P02549 | SPTA1 | -1.11 | 1.41E-03 |
| P01860 | IGHG3 | -1.22 | 6.48E-03 |
| P02649 | APOE | -1.35 | 2.05E-15 |
| P01817 | IGHV2-5 | -1.37 | 7.34E-03 |
| P02671 | FGA | -1.51 | 2.80E-41 |
| P61769 | B2M | -1.64 | 1.85E-03 |
| P02655 | APOC2 | -1.65 | 7.91E-04 |
| P01714 | IGLV3-1 | -1.88 | 6.11E-03 |
| P02788 | LTF | -2.07 | 2.92E-03 |
| P01705 | IGLV2-23 | -2.08 | 5.44E-03 |
| P01861 | IGHG4 | -2.12 | 2.58E-05 |
| P05090 | APOD | -2.19 | 1.79E-11 |
| P02745 | C1QA | -2.57 | 2.05E-15 |
| P02652 | APOA2 | -2.59 | 7.52E-27 |
| Q6Q788 | APOA5 | -2.80 |  |
| P35542 | SAA4 | -3.74 | 8.51E-15 |
| P0DJI8 | SAA1 | -∞ | - |
| Q04756 | HGFAC | -∞ | - |
| P05452 | CLEC3B | -∞ | - |
| P11597 | CETP | -∞ | - |
| O00592 | PODXL | -∞ | - |
| Q86YZ3 | HRNR | -∞ | - |
| Q9NZP8 | C1RL | -∞ | - |
| Q9Y6Z7 | COLEC10 | -∞ | - |
| P61981 | YWHAG | -∞ | - |
| P15311 | EZR | -∞ | - |
| Q6UXB8 | PI16 | -∞ | - |
| P80723 | BASP1 | -∞ | - |
| P22352 | GPX3 | -∞ | - |
| P31146 | CORO1A | -∞ | - |
| P31946 | YWHAB | -∞ | - |
| P63218 | GNG5 | -∞ | - |
