# Supplementary material for: Phenotypic and proteomic analysis of plasma extracellular vesicles highlights them as potential biomarkers of primary Sjögren syndrome
Source: Front Immunol. 2023 Jul 17;14:1207545. doi: 10.3389/fimmu.2023.1207545 (PMC10388367; doi:10.3389/fimmu.2023.1207545)
Supplement: Supplementary file 4 [file Table_4.docx]

Table S4 | **Molar percentages of** **differentially expressed proteins in pSS vs HD and/or SLE**

Protein molar percentages (Mol percent.) for each conditions (SLE, pSS, HD). Mol percent. were estimated using the intensity-Based Absolute Quantification (iBAQ) method (23). Common upregulated proteins with pSS vs HD comparison are highlighted in red, immunoglobulin proteins are highlighted in grey. pSS, primary Sjogren syndrome; DEP, differentially expressed proteins; SLE, systemic lupus erythematosus.

| **Protein** | **Gene** | **Mol percent. (%)** | | |
| --- | --- | --- | --- | --- |
|  |  | **HD** | **SLE** | **pSS** |
| A0A075B6H9 | IGLV4-69 | 0,03152 | 0,03807 | 0,17468 |
| A0A075B6I1 | IGLV4-60 | 0,00309 | 0,00449 | 0,06095 |
| A0A0C4DH31 | IGHV1-18 | 0,01342 | 0,03120 | 0,08121 |
| A0A0G2JRQ6 | A0A0G2JRQ6 | 0,01552 | 0,09187 | 0,03564 |
| P01599 | IGKV1-17 | 0,00989 | 0,01847 | 0,04143 |
| P01619 | IGKV3-20 | 0,05425 | 0,02939 | 0,20460 |
| P01700 | IGLV1-47 | 0,41190 | 0,17627 | 3,19600 |
| P01854 | IGHE | - | 0,00230 | 0,00158 |
| P01857 | IGHG1 | 4,32760 | 7,33520 | 15,39200 |
| P01880 | IGHD | 0,00301 | 0,00610 | 0,01448 |
| P02766 | TTR | 0,04848 | 0,08817 | 0,13448 |
| P0DTE1 | IGHV3-38-3 | 0,02488 | 0,01040 | 0,11311 |
| P11215 | ITGAM | - | 0,00074 | 0,00061 |
| P16671 | CD36 | 0,00346 | 0,00254 | 0,00146 |
| P20742 | PZP | 0,01745 | 0,00974 | 0,02386 |
| P29622 | SERPINA4 | 0,00181 | 0,00384 | 0,00921 |
| P35443 | THBS4 | 0,00103 | 0,00100 | 0,00315 |
| P61026 | RAB10 | 0,00124 | 0,00426 | 0,00820 |
| P80748 | IGLV3-21 | 0,00792 | 0,01392 | 0,03791 |
| Q15582 | TGFBI | 0,00042 | 0,00105 | 0,00203 |
| Q6UX06 | OLFM4 | 0,00081 | 0,00050 | 0,00279 |
| Q9NQ79 | CRTAC1 | 0,00047 | 0,00134 | 0,00054 |
| A0A075B6I0 | IGLV8-61 | 0,08093 | 0,04692 | 0,02502 |
| A0A075B7B8 | IGHV3OR16-12 | 0,29433 | 0,20302 | 0,03322 |
| A0A0B4J1Y9 | ND4L | 0,06340 | 0,02909 | 0,04007 |
| A0A0J9YX35 | IGHV3-64D | 0,10036 | 0,05578 | 0,10594 |
| P01591 | JCHAIN | 2,43370 | 1,14570 | 0,89873 |
| P01601 | IGKV1D-16 | 0,00679 | 0,00782 | 0,01106 |
| P01602 | IGKV1-5 | 0,72799 | 0,52273 | 0,07646 |
| P01703 | IGLV1-40 | 0,13821 | 0,13802 | 0,90866 |
| P01705 | IGLV2-23 | 0,13676 | 0,11437 | 0,01225 |
| P01714 | IGLV3-1 | 0,26109 | 0,49658 | 0,03997 |
| P01764 | IGHV3-23 | 0,01330 | 0,00462 | 0,00822 |
| P01780 | IGHV3-7 | 0,56984 | 0,41202 | 0,21807 |
| P01817 | IGHV2-5 | 0,01486 | 0,01592 | 0,01974 |
| P01834 | IGKC | 3,33890 | 3,02630 | 7,73720 |
| P01859 | IGHG2 | 0,83902 | 0,51492 | 0,10193 |
| P01860 | IGHG3 | 0,96157 | 1,11660 | 0,28915 |
| P01861 | IGHG4 | 0,11539 | 0,10319 | 0,00699 |
| P01877 | IGHA2 | 0,03017 | 0,01085 | 0,03283 |
| P04430 | IGKV1-16 | 0,04173 | 0,03253 | 0,10635 |
| P04432 | IGKV1D-39 | 0,02496 | 0,01175 | 0,02797 |
| B0I1T2 | MYO1G | 0,00149 | 0,00273 | 0,00038 |
| O00560 | SDCBP | 0,00242 | 0,00200 | 0,00309 |
| O00592 | PODXL | 0,00331 | 0,00256 | 0,00057 |
| O14745 | SLC9A3R1 | 0,00191 | 0,00086 | 0,00260 |
| O15400 | PLXNB2 | 0,00100 | 0,00148 | 0,00107 |
| O43866 | CD5L | 0,90933 | 0,53463 | 0,23465 |
| O75636 | FCN3 | 0,23194 | 0,26920 | 0,08778 |
| O75955 | FLOT1 | 0,00094 | 0,00054 | 0,00056 |
| O95445 | APOM | 0,08543 | 0,05339 | 0,01405 |
| P00558 | PGK1 | 0,00053 | 0,00076 | 0,00079 |
| P00739 | HPR | 2,15140 | 2,48790 | 1,33400 |
| P00748 | F12 | 0,00292 | 0,00459 | 0,00840 |
| P00915 | CA1 | 0,00333 | 0,00176 | 0,00325 |
| P01023 | A2M | 6,30520 | 5,54360 | 6,01250 |
| P02042 | HBD | 0,01191 | 0,00111 | - |
| P02538 | KRT6A | 0,00167 | 0,00107 | 0,00181 |
| P02549 | SPTA1 | 0,00351 | 0,00096 | 0,00020 |
| P02647 | APOA1 | 2,54180 | 2,54730 | 0,43991 |
| P02649 | APOE | 0,40005 | 0,48219 | 0,11420 |
| P02652 | APOA2 | 0,53488 | 0,48986 | 0,24926 |
| P02654 | APOC1 | 0,12970 | 0,12656 | 0,00868 |
| P02655 | APOC2 | 1,63390 | 0,71619 | 0,05053 |
| P02656 | APOC3 | 0,04536 | 0,02000 | 0,01391 |
| P02671 | FGA | 2,93170 | 6,03220 | 0,72534 |
| P02730 | SLC4A1 | 0,03896 | 0,01515 | 0,00823 |
| P02745 | C1QA | 0,40580 | 0,44327 | 0,03458 |
| P02749 | APOH | 0,07175 | 0,09430 | 0,02171 |
| P02768 | ALB | 4,39600 | 5,36080 | 0,89416 |
| P02775 | PPBP | 0,03113 | 0,01127 | 0,06939 |
| P02776 | CXCL4 | 0,10425 | 0,07428 | 0,27479 |
| P02787 | TF | 0,90038 | 0,88954 | 0,22497 |
| P02788 | LTF | 0,00182 | 0,00972 | 0,00062 |
| P02790 | HPX | 0,16158 | 0,17103 | 0,08767 |
| P04004 | VTN | 0,04778 | 0,12194 | 0,02529 |
| P04083 | ANXA1 | 0,00072 | 0,00077 | - |
| P04180 | LCAT | 0,00341 | 0,00115 | 0,00039 |
| P04275 | VWF | 0,51238 | 0,52666 | 0,33202 |
| P04406 | GAPDH | 0,01361 | 0,01326 | 0,04590 |
| P05090 | APOD | 0,19543 | 0,27928 | 0,06531 |
| P05155 | SERPING1 | 0,06173 | 0,03163 | 0,01104 |
| P05362 | ICAM1 | 0,00038 | 0,00042 | 0,00062 |
| P05452 | CLEC3B | 0,00542 | 0,01449 | 0,00155 |
| P06733 | ENO1 | 0,00437 | 0,00318 | 0,00128 |
| P07195 | LDHB | 0,00267 | 0,00213 | 0,00441 |
| P07355 | ANXA2 | 0,00304 | 0,00266 | 0,00117 |
| P08185 | SERPINA6 | 0,00405 | 0,00223 | 0,01103 |
| P08754 | GNAI3 | 0,00066 | - | - |
| P09172 | DBH | 0,00061 | 0,00074 | 0,00219 |
| P0C0L4 | C4A | 0,15816 | 0,16190 | 0,14332 |
| P0DJI8 | SAA1 | 0,00678 | 0,01274 | 0,00285 |
| P0DP25 | CALM3 | 0,00358 | 0,00436 | 0,00100 |
| P11166 | SLC2A1 | 0,00395 | - | - |
| P11277 | SPTB | 0,00293 | 0,00130 | 0,00022 |
| P11597 | CETP | 0,00175 | 0,00267 | 0,00117 |
| P13224 | GP1BB | 0,02067 | 0,01223 | 0,00238 |
| P13987 | CD59 | 0,03283 | 0,02053 | 0,00269 |
| P14151 | SELL | 0,00193 | 0,00096 | - |
| P14618 | PKM | 0,00443 | 0,00208 | 0,00087 |
| P15311 | EZR | 0,00241 | 0,00114 | 0,00043 |
| P16070 | CD44 | 0,00191 | 0,00102 | 0,00221 |
| P17858 | PFKL | 0,00011 | 0,00051 | 0,00136 |
| P17936 | IGFBP3 | 0,00433 | 0,00205 | 0,00102 |
| P18428 | LBP | 0,00671 | 0,01038 | 0,00143 |
| P19652 | ORM2 | 0,09820 | 0,09859 | 0,27140 |
| P20851 | C4BPB | 0,31057 | 0,29590 | 0,11214 |
| P21926 | CD9 | 0,00097 | 0,00299 | - |
| P22352 | GPX3 | 0,00141 | 0,00423 | - |
| P24821 | TNC | 0,00224 | 0,00183 | 0,00534 |
| P26038 | MSN | 0,00995 | 0,00240 | 0,00063 |
| P26927 | MST1 | 0,00059 | 0,00049 | 0,00109 |
| P27918 | CFP | 0,03931 | 0,02182 | 0,00207 |
| P31146 | CORO1A | 0,00265 | 0,00130 | - |
| P31946 | YWHAB | 0,00142 | 0,00209 | - |
| P35527 | KRT9 | 0,05533 | 0,03344 | 0,01020 |
| P35542 | SAA4 | 0,16430 | 0,44463 | 0,01195 |
| P35908 | KRT2 | 0,06772 | 0,03734 | 0,00709 |
| P51884 | LUM | 0,00283 | 0,00715 | 0,00412 |
| P52566 | ARHGDIB | 0,00250 | 0,00258 | 0,00179 |
| P55056 | APOC4 | 0,11081 | 0,04705 | 0,01592 |
| P60660 | MYL6 | 0,00853 | 0,01426 | 0,01027 |
| P61225 | RAP2B | 0,00198 | 0,00093 | 0,00118 |
| P61769 | B2M | 0,05239 | 0,07347 | 0,00493 |
| P61981 | YWHAG | 0,00130 | 0,00173 | 0,00026 |
| P62879 | GNB2 | 0,01245 | 0,00684 | 0,00181 |
| P63104 | YWHAZ | 0,02034 | 0,01617 | 0,00760 |
| P63218 | GNG5 | 0,00623 | 0,00862 | - |
| P68871 | HBB | 1,81700 | 0,37821 | 0,32590 |
| P69905 | HBA1 | 1,48730 | 0,28618 | 0,36432 |
| P80723 | BASP1 | 0,00238 | 0,00225 | - |
| Q04756 | HGFAC | 0,00120 | 0,00154 | 0,00175 |
| Q06830 | PRDX1 | 0,00164 | 0,00172 | 0,00075 |
| Q13093 | PLA2G7 | 0,00060 | 0,00044 | 0,00062 |
| Q13790 | APOF | 0,00502 | 0,00205 | 0,00247 |
| Q15485 | FCN2 | 0,02048 | 0,01689 | 0,00482 |
| Q15599 | SLC9A3R2 | 0,00256 | 0,00143 | 0,00038 |
| Q15833 | STXBP2 | 0,00023 | 0,00073 | 0,00046 |
| Q658P3 | STEAP3 | 0,00075 | 0,00033 | 0,00046 |
| Q6Q788 | APOA5 | 0,01180 | 0,01356 | 0,00285 |
| Q6UXB8 | PI16 | 0,00116 | 0,00199 | - |
| Q86YZ3 | HRNR | 0,00044 | 0,00028 | 0,00011 |
| Q8WUA8 | TSKU | 0,01386 | 0,01406 | 0,00208 |
| Q96IY4 | CPB2 | 0,00228 | 0,00219 | 0,00115 |
| Q96KN2 | CNDP1 | 0,00101 | 0,00066 | 0,00029 |
| Q96Q89 | KIF20B | 0,00048 | 0,00061 | 0,00028 |
| Q9BYG3 | NIFK | 0,00093 | 0,00216 | 0,00230 |
| Q9H4G4 | GLIPR2 | 0,00559 | 0,00155 | 0,00202 |
| Q9NZP8 | C1RL | 0,00224 | 0,00321 | 0,00075 |
| Q9Y624 | F11R | 0,00201 | 0,00179 | - |
| Q9Y696 | CLIC4 | 0,00126 | 0,00108 | 0,00091 |
| Q9Y6Z7 | COLEC10 | 0,00211 | 0,00198 | 0,00095 |
